# Routine Health Information Utilization and Associated Factors among Health Professionals Working in Public Health Facilities of the South Region, Ethiopia

**DOI:** 10.4314/ejhs.v32i2.24

**Published:** 2022-03

**Authors:** Sewunet Sako, Girma Gilano, Yilma Chisha, Misgun Shewangizaw, Teshale Fikadu

**Affiliations:** 1 Department of Health Informatics, School of Public Health, College of Medicine and Health Science, Arba Minch University, Arba Minch, Ethiopia; 2 Department of Public Health, School of Public Health, College of Medicine and Health Science, Arba Minch University, Arba Minch, Ethiopia

**Keywords:** Routine health information utilization, public health facilities, Southern Ethiopia

## Abstract

**Background:**

Routine health information is the pillar for planning and management of health services and plays a vital role in effective and efficient health service delivery, decision making, and the improvement of programs. Therefore, this study aimed to assess routine health information utilization and associated factors among health professionals working in public health facilities of the south region.

**Methods:**

Institution based cross-sectional study design was employed. Data was collected from randomly selected 719 participants using a pre-tested, interviewer administered structured questionnaire. Bivariate and multivariate logistic regression analyses were carried out.

**Result:**

The overall utilization of routine health information was 63.1. Place of residence, HMIS personnel, HMIS code, overwhelming data source, population based data, data quality control, feedback, monitoring chart, 8.467) and data transfer policy were factors significantly associated with utilization of routine health information.

**Conclusion:**

Six out of ten health professionals had utilized routine health information. Place of residence, HMIS personnel, HMIS code, overwhelming data source, population based data, data quality control, feedback, monitoring chart and data transfer policy had significant associations with routine health information utilization. Therefore, concerned health authorities need to work on these factors to improve the utilization.

## Introduction

WHO defines Health Information System (HIS) as a system that integrates data collection, processing, reporting, and use of the information necessary for improving health service effectiveness and efficiency through better management at all levels of health system (1). The main objective of a health information system is to generate quality information that health system stakeholders can use for making evidence-based decisions (2).

Data from public, private, and community-level health facilities and health organizations produced in systematic intervals encompass routine health information systems (RHIS). These data provide an image of health status, health services, and health resources (3). RHISs can serve as the backbone for planning, decisionmaking, resource distribution and designing strategy among periphery, intermediate and central levels of the health system (4).

It is also the pillar for planning and management of health services at various levels of the health system as it can play a vital role in effective and efficient service delivery, decision making, and the improvement of programs. Assessing effectiveness and efficiency of health service delivery help us to know how well primary healthcare services meet the health needs of the population. And this is achieved through collecting, processing and analyzing a series of performance indicators captured mainly through RHIS (5,6).

Health data quality has its own benefits for patients, health institutions and researchers. Patients are more likely to get better and safer care if healthcare providers have access to correct and consistent patient data like results of investigations, information on allergies, past medical history, and potential drug interactions. Similarly, health institutions require high quality health data to provide quality health services for their clients. Likewise, quality data help researchers to investigate and provide evidence that can support particular care processes and beyond (7). However, decisions that are not evidence-based results in failures of many health programs (8–10). The ultimate objective of a RHIS is to ensure that health information is used rationally, effectively and efficiently to improve health action. Accurate, complete and timely health information are used to identify strengths and gaps of the health system functions and services and accordingly to take actions that improves performance. Also, it is important for health managers to advocate for possible intervention and policy amendment for concerns that are out of their control. In that, it helps the managers and health professionals to confirm whether they are doing things right and doing the right things or not (9,11,12).

The study conducted in the North Shewa zone reported that more than two-thirds of the managers utilized routine health information for decision making particularly for human deployment, to know physician per capita, pharmacist per capita, laboratory professional per tests as compared to the standards, for resource allocation, and for quality improvement (12).

The key components of health data quality and statistical reports are accuracy and validity, consistency, completeness, timeliness, legibility, accessibility, confidentiality, precision, integrity, and relevance (7). More specifically data quality is measured using the four core dimensions: accuracy, completeness, timeliness and relevance. Accuracy of health data is measured by comparing data between facility records and reports, and between facility reports and administrative area databases, respectively. Completeness is measured in two ways: filling in all data elements in the report form, and total number of reports received out of expected number of reports from all reporting health facilities under the administrative area. A report is considered submitted timely if it is submitted before an accepted deadline. Relevance of the data is assessed by comparing data collected against management information needs (7,9).

Since 2008, there is visible improvement in knowledge and understanding of the role of routine health information on health systems of Ethiopia and therefore, substantial human and financial resources have been devoted to the collection of health data from institution and community based sources (13). Nevertheless, use of the organized, accurate, consistent and complete information for aforementioned activities is still very weak within the health sector. This in turn resulted in the failure of many health programs, as most executive decisions are being made deprived of evidence (6,14–18).

Literature disclosed that the main concerns to improve evidence-based decision making include sound demand of health data, its collection and analysis, making it available for decision makers, and promoting its use (9,15,19). However, poor data quality, poor access to data, lack of capacity of health managers and providers in core competencies for data use, centralization and fragmentation of health information systems and poor identification of information needs remain the major barriers in the developing countries for translating data into action (5).

According to the findings from the studies conducted in different parts of the country, use of routine health information for decision making remains low. The level of routine health information utilization among health professionals working in public health facilities was found to be highest (78.5%) in North Gondar (15), 69.3% in Hadiya zone (14), 71.6% in North Shewa (12) and lowest (45.8%) in East Gojjam Zone (6).

In consideration of the above facts, the country has been intensely dedicated to reinforce its national HIS through taking different actions. The Federal Ministry of Health (FMOH) of Ethiopia encompassed information revolution as one of the four transformation agendas in the health sector transformation plan (20). Information revolution involves a drastic shift from traditional methods of information use to a methodical data management approach run by a corresponding level of technology (21).

Several studies stated that the core determinants of routine health information utilization are technical, behavioral and organizational factors (6,14–16). According to these studies, factors that were significantly associated with routine health information utilization include knowledge and skills required for data processing, data analysis, data interpretation and problem-solving, supportive supervision and feedback, organizational infrastructure, HMIS training, computer skill, and availability of HMIS resources like human resources, guidelines and formats.

However, less is known about evidence-based actions to successively progress the use of information for decision-making around improving quality, effectiveness, and efficiency of health service delivery despite significant attention given by the Ministry of Health of Ethiopia to the routine health information data quality improvement. Furthermore, various studies have been conducted in different parts of the country, but no region wide study was conducted in Ethiopia. Therefore, this study aimed to assess routine health information utilization and associated factors among health professionals working in public health facilities of the south region, southern Ethiopia.

## Materials and Methods

**Study design and setting**: An institution-based cross-sectional study was conducted at public health facilities of the south region from January 2018 to June 2018. The region is one of the largest regions in Ethiopia which covers 118,000 square kilometers. Hawassa, the capital city of the region, is located at 272 km south of Addis Ababa, the capital of Ethiopia. During the year of this study, there were 51 hospitals (referral, general and primary), and 732 health centers (783 total health facilities) in the region.

**Study participants, sample size, and sampling procedure**: The sample size was determined using the single population proportion formula, assuming 69.3% prevalence of routine health information utilization in the Hadiya Zone (14), 95% level of confidence, 5% margin of error, design effect of 2, and 10% non-response rate. Based on the above assumptions, the sample size calculated was 719. There were 15 zones and 4 special woredas in the region during the study period. Out of these clusters, five were randomly selected using a lottery method. All the health professionals working in the region were the source population for this study. And total health professionals working in public health facilities of the selected five clusters were taken as a study population. Then, proportional allocation technique was used to select 719 health professionals working in the selected clusters. Lastly, a simple random sampling method was used to select health professionals from each selected public health facility for an interview.

**Measurement of variables**: The response variable of the study was utilization of routine health information (yes or no). It was assessed by using information for decision making, to take immediate action such as feedback from respective supervisors, calculation of area coverage, presentation of key indicators with charts or tables and presentation of achievements of targets. Those health professionals who practiced minimum three of the above criteria were considered as health professionals utilized routine health information else not (18).

Whereas the independent variables of the study were socio-demographic variables (respondent's sex, age, educational status, work experience, place of residence, monthly salary), HMIS input variables (availability of trained/skilled staff, presence of guideline concerning HMIS use, office for HMIS, presence of HMIS personnel, equipment for HMIS, trained on basic computer literacy, in-service training on HMIS), HMIS processes variables (collect health data, user friendly tool, assign HMIS code, compile HMIS report, conduct regular local data quality control, use appropriate technology, conduct supportive supervision, receive feedback, presence of meeting minutes) and HMIS output and utilization (Map of catchment area, catchment population profile, unit staff profile, outreach locations & schedule, ten top causes of morbidity, ten top causes of morbidity in < 5, ten top causes of mortality in Hospital, Reproductive health (ANC and skilled attendant delivery), immunization monitoring for under 1, disease cases (Malaria all ages, and Pneumonia amongst Under 1s), HIV/AIDS (VCT, PMTCT, and ART), OPD attendance, inpatient admission, average length of stay, bed occupancy, area coverage indicators, organizational chart, area coverage indicators, performance monitoring chart, accessibility of health data, incentives for use of health information, data security guideline, data transfer guideline, and any mechanism to disseminate heath information).

The questionnaire was adapted from RHIS Performance Diagnostic tool of PRISM framework (9). Data were collected using the pretested, structured, and interviewer administered questionnaire. A two-days training was given for twelve data collectors and four supervisors on the objective of the study and the confidentiality of information. Both data collectors and supervisors were recruited depending on their previous exposure of data collection and their research related activities.

Also a checklist was developed to collect HMIS output and utilization data based on “Information use guideline and display tools: HMIS/M&E technical standards: Area 4” of FMOH that specifies minimum display charts to be maintained by health institutions (22).

**Data processing and analysis**: Data was entered into Epi-info version 7 and exported to the STATA version 14 for further analysis. To explain the study population in relation to relevant variables descriptive statistics was used. Variables with a p-value of less than 0.25 in the bivariate analysis were entered into the multivariate logistic regression analysis. Both Crude Odds Ratio (COR) and Adjusted Odds Ratios (AOR) with 95% confidence intervals were estimated to show the strength of associations. Finally, a p-value of less than 0.05 in the multivariate logistic regression analysis was used to identify variables significantly associated with the utilization of routine health information.

**Ethics Approval**: Ethical clearance was obtained from the Ethical Review Board of the Arba Minch University. A letter of permission was obtained from Gamo Gofa, Wolaita, South Omo, Kambata Tambaro and Gedeo Zonal health departments. Verbal consent was obtained from each participant before participation in the research process. Furthermore, privacy and confidentiality of information was strictly guaranteed by all data collectors and investigators. The information retrieved was used only for the study.

## Results

**Socio-demographic characteristics**: Out of 783 health facilities available in the region, 177 health facilities (health center and hospitals) were included in the study. Within these health facilities a total of 708 health professionals were involved in the study, giving a response rate of 98.5%. From the total health professionals participated in the study, more than half (58.2%) of the respondents were males and the remaining 296(41.8%) were females. A bit higher than half (53.7%) of the respondents were in the age range of 25–29 and nearly three fourth (71.7%) were diploma holders. Majority of the respondents (84.9%) have a salary of >2800 ETB and 380(53.7%) of them have less than 5 years of service (Table 1).

**Table 1:** Socio-demographic characteristics of respondents working at public health facilities of South region of Ethiopia, 2018/19

Variables	Frequency	Percent
Type of the health facility		
Health Center	667	94.2
Hospital	41	5.8
Unit/department		
OPD	519	73.3
IPD	19	2.7
MCH	157	22.2
Other	13	1.8
Sex		
Male	412	58.2
Female	296	41.8
Age (in years)		
20–24	159	22.5
25–29	380	53.7
30–34	110	15.5
35–39	28	3.9
40–44	20	2.8
≥45	11	1.6
Educational status		
Grade 9–12	23	3.3
Diploma	508	71.7
Degree	177	25.0
Work experience (in years)		
<5	380	53.7
5–10	278	39.3
≥11	50	7.0
Place of residence		
Urban	436	61.6
Rural	272	38.4
Monthly salary (in birr)		
700–1200	6	0.8
1201–1600	11	1.6
1601–2000	14	2.0
2001–2400	25	3.5
2401–2800	51	7.2
>2800	601	84.9

**HMIS Input**: This study has shown that the necessity of HMIS data for planning, decision making and for the various routine tasks by almost all health professionals (98.5%) have been increased. Regarding the outcome of the HMIS implementation except tenth of the respondents, the remaining (92.2%) agree that it has brought change in their health facility. As a result, nearly the same number of individuals (92.1%) have reported as they feel comfort while recording, processing, analyzing and disseminating HMIS data.

Out of all the respondents, 250(35.3%) have revealed that there is no trained/skilled staff able to fill out formats (HMIS tools) in their respective unit/department. Among the total participants of the study, about 380(53.7%) have assured that there was a legislative, regulatory and planning framework concerning the use of HMIS. Half of the respondents (49.6%) approved that there is no office assigned specifically to HMIS and 587(82.9%) respondents confirmed the presence of HMIS focal person in their health facility.

**Figure 1:**
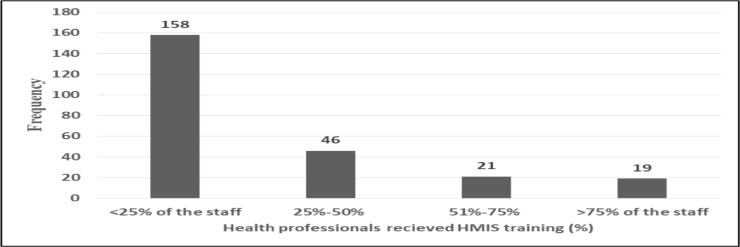
Number of health professionals received in-service training on HMIS in the last 12 months, South Ethiopia, 2018/19

The majority of the respondents (94.4%) replied that their facility did not assign a budget specifically for HMIS activities. It was found that availability of necessary equipment for HMIS activities were reported by 638(90.1%) respondents. According to the study, basic computer literacy training was not given for nearly two third (63.1%) of the study participants. And also only 244(34.5%) health professionals took inservice training on HMIS activities (Figure 1) (Table 2).

**Table 2:** Availability of HMIS input at public health facilities of South region of Ethiopia, 2018/19

Variable	Frequency	Percent
Have trained/skilled staff		
No	250	35.3
Yes	458	64.7
Guideline concerning HMIS use available:		
No	380	53.7
Yes	328	46.3
Office assigned specifically to HMIS:		
No	351	49.6
Yes	357	50.4
Have HMIS focal person		
No	121	17.1
Yes	587	82.9
Assign budget for HMIS activities		
No	668	94.4
Yes	40	5.6
Necessary equipment for HMIS available:		
No	70	9.9
Yes	638	90.1

**HMIS process**: Almost all (97.8%) of the respondents described that clinical data collection using HMIS tools available in their unit was taken as part of their routine activity of patient care. But after each clinical visit only 276(39.0%) of all the respondents give HMIS code for the collected clinical data. The majority (86.6%) of respondents revealed that HMIS tools for data collection and reporting formats were user friendly. According to the present study, nearly two-third of the respondents (64.7%) answered that the sources of data to be collected are overwhelming.

Lower than half of the respondents (45.3%) agreed that their facility uses population based data to obtain denominator data for the HMIS indicators. Regarding compilation of HMIS reports, nearly one fifth of the respondents (19.1%) reported that they did not have such experience. Three fourth (75.6%) of the respondents accepted that the reformed HMIS records were easily accessible for the authorized personnel. Concerning the simplicity of the procedure for reporting HMIS data, 505(71.3%) respondents approved that the procedure was clear for them.

From the total respondents, 322(45.5%) did know a rule for minimum period of HMIS records retaining time in their health facility. Whereas, two-third of the study participants (66.1%) reported that self-assessment mechanisms (like Lot Quality Assurance Sampling) are in place to control the quality of HMIS data. The utilization of appropriate technology such as calculators, software and databases for HMIS data analysis, transfer and presentation was practiced by 305(43.1%) health professionals working in the health facilities of the region.

**Figure 2:**
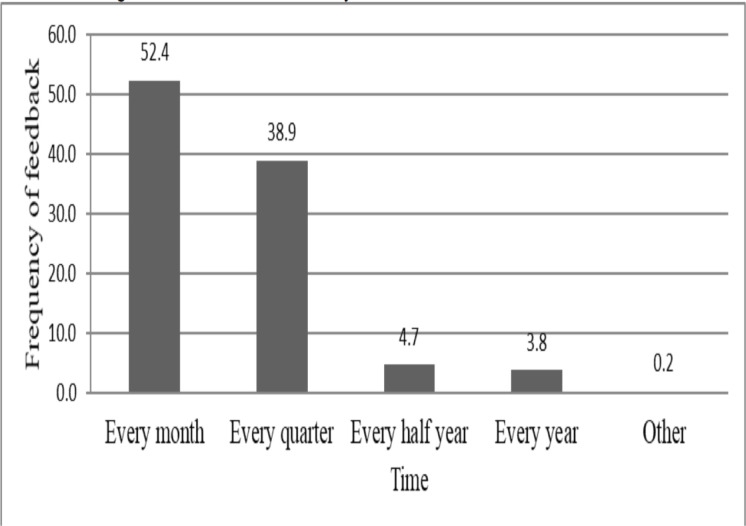
Frequency of feedback received by health professionals working in public health facilities of South region on the last two supervisory visits, 2018/19.

Despite the fact that nearly one-fifth (20.1%) of the respondents were not supervised, surprisingly more than half (52.4%) of the supervised respondents were supervised every month (Figure 2). Among the supervised respondents 471(83.2%) have received regular feedback from the next higher health authority (Table 3).

**Table 3:** HMIS process in the selected public health facilities of South region, 2018/19

Variable	Frequency (#)	Frequency (%)
User friendly data collection and reporting format		
No	95	13.4
Yes	613	86.6
HMIS code given just after each clinical visit		
No	432	61.0
Yes	276	39.0
Data sources to be collected are overwhelming		
No	250	35.3
Yes	458	64.7
The facility compile HMIS report		
No	135	19.1
Yes	573	80.9
Conduct regular local data quality control		
No	240	33.9
Yes	468	66.1
Use appropriate technology for data management		
No	403	56.9
Yes	305	43.1
Supportive supervision given for your unit/facility		
No	142	20.1
Yes	566	79.9
Received feedback		
No	95	16.8
Yes	471	83.2
How frequently do you get the feedback		
Every month	247	52.4
Every quarter	183	38.9
Every half year	22	4.7
Every year	18	3.8
Other	1	0.2

**HMIS output and utilization**: Less than one third (29.7%) of the health professionals depict their achievements using a performance monitoring chart that was displayed in their units. A bit higher than a quarter (27.7%) of health professionals who appropriately use health information for their routine tasks were motivated by the managers of their respective health facilities to promote culture of information use.

The availability of policy/guideline/standard in the facility for information use, data security and transfer of data between the facility and external users of the records were reported to be 307(43.7%), 314(44.4%) and 270(38.1%) respectively. Less than half (43.4%) of the health professionals reported that their health facilities have mechanisms to disseminate health information outside the health facilities aside from the reports sent to the health offices. Based on the set criteria, 447(63.1%) health professionals working in the public health facilities of the region utilized routine health information.

**Factors associated with routine health information utilization**: In the bivariate logistic regression analysis place of residence, presence of skilled staff, availability of HMIS personnel, trained basic computer literacy, trained in-service HMIS training, assigning HMIS code, overwhelming data source, use population based data, compile HMIS report, conduct local data quality control, conduct supportive supervision, provide feedback, data consistency, posted performance monitoring chart, have data security policy, have data transfer policy and have any mechanism for health information dissemination were factors associated with routine health information utilization at a p-value of less than 0.25.

Based on the multivariate logistic regression analysis place of residence, availability of HMIS personnel, assigning HMIS code, overwhelming data source, use population based data, conduct local data quality control, receive feedback, posted performance monitoring chart and have data transfer policy showed significant association with routine health information utilization at a p-value of less than 0.05 (Table 4).

**Table 4:** Factors associated with routine health information utilization among health professionals working in public health facilities of South region, 2018/19

Variable	Utilization status		Crude OR(95% CI)	Adjusted OR(95% CI)	p-value

	Utilized	Not utilized			
Place of residence					
Urban	291	145	1.49 (1.092, 2.039)	2.04(1.315,3.153)	0.001*
Rural	156	116	1	1	
Is there skilled staff					
Yes	266	192	0.53 (0.378, 0.737)	1.20 (0.707, 2.044)	
No	181	69	1	1	0.496
HMIS personnel available:					
Yes	356	231	0.51(0.326,0.793)	2.25(1.157,4.393)	
No	91	30	1	1	0.017*
Trained basic computer literacy					
Yes	150	111	0.68 (0.498, 0.935)	1.35 (0.754, 2.424)	
No	297	150	1	1	0.311
Trained HMIS					
Yes	119	125	2.53 (1.838, 3.492)	1.51(0.816,2.809)	
No	328	136	1	1	0.188
HMIS code given					
Yes	129	147	3.18(2.311,4.373)	2.07 (1.292, 3.309)	
No	318	114	1	1	0.002*
Overwhelming data source					
Yes	266	192	1.89(1.356,2.643)	2.43 (1.506, 3.914)	<0.001*
No	181	69	1	1	
Use population based data					
Yes	171	150	2.18(1.598,2.976)	1.67 (1.052, 2.647)	
No	276	111	1	1	0.030*
Compile HMIS report					
Yes	349	224	1.70(1.124,2.571)	1.15(0.595,2.238)	
No	98	37	1	1	0.672
Are there local quality control					
Yes	263	205	2.56 (1.804, 3.636)	3.36(1.918,5.889)	
No	184	56	1	1	<0.001*
Received feedback					
Yes	264	207	2.45 (1.484, 4.061)	3.11(1.573,6.140)	
No	72	23	1	1	0.001*
Is there monitoring chart					
Yes	95	115	2.92 (2.091, 4.073)	5.19(3.177,8.467)	
No	352	146	1	1	<0.001*
Have data transfer policy					
Yes	188	82	1.59(1.148,2.186)	4.17(1.796,9.697)	
No	259	179	1	1	0.001*

* Variable significant at p-value less than 0.05

## Discussion

Health management information system (HMIS) is an information system specially designed to assist in the management and planning of health programmes, as opposed to delivery of care (1). Using the health information delivered through the HMIS for action-oriented performance monitoring, particularly where the data is produced, is the main objective of the HMIS/M&E process. Therefore, this study was designed to assess routine health information utilization and its associated factors in public health facilities of the south region.

The overall utilization of routine health information in the study area was 63.1% (95% CI: 59.6% – 66.67%). This finding is lower as compared to the study conducted at Hadiya zone and North Gondar which was 69.3% and 78.5% respectively (15,23). The decrease in this study might be due to differences in operational definition of routine health information utilization and differences in the study period. In contrast, the level of routine health information utilization in our study was higher than the study reported from East Gojjam zone, Western Amhara, Dire Dawa, and East Wollega which was below 45.8%, 38.4%, 53.1% and 57.9% respectively (6,16,17,24). The possible explanations for this variation could be differences in criteria used for routine health information utilization and facility type; woreda health offices, hospitals, health centers and health posts were included in their study and used only activities of performance review team /PRT/ to assess utilization of data but in our case, only health centers and hospitals were considered and instead of PRT we considered every health workforce.

In this study, the expected odds of routine health information utilization for urban respondents was 2.04 times more likely as compared to rural respondents, given the other conditions constant [AOR=2.04, 95% CI: (1.315, 3.153), p-value=0.001]. This finding is in agreement with the finding of a study conducted in Western Amhara (17). The possible reason for this discrepancy might be shortage of HMIS tools due to transport inaccessibility, inadequate supervision and lack of skilled staff in the rural set up.

According to the multivariate logistic regression analysis, the expected odds of routine health information utilization for health professionals working in health facilities which have personnel specifically assigned to HMIS was 2.25 times more likely as compared to those facilities which have no personnel specifically assigned to HMIS, given the other conditions constant [AOR=2.25, 95% CI: (1.157, 4.393), p-value=0.017]. This finding is supported by the study findings from North Gondar (15), East Gojjam zone (6), Dire Dawa (16) and Western Amhara (17). This could be because the presence of experts who have good knowledge and skill on how to collect, process, analyze, disseminate and use the routine health information for decision making, plan setting and for routine daily activities motivates other health professionals to take the same action. And also, the expert may enhance routine health information utilization by providing relevant recording and reporting tools for each unit of the facility, mentoring health professionals working there and by monitoring the use of provided tools for the intended purpose.

Assigning HMIS code after each clinical visit is important to provide better data for evaluating and improving the quality of patient care. HMIS code is given based on a national disease classification system designed according to diagnostic capability of the country (25). In the present study, the expected odds of routine health information utilization was 2.07 times higher among health professionals who had given HMIS code after each clinical visit as compared to their counterparts, given the other conditions constant [AOR=2.07, 95% CI: (1.292, 3.309), p-value= 0.002].

Literatures reported that correct, reliable, complete and timely health information is crucial for evidence based decision-making (26). In this study, the expected odds of routine health information utilization among health professionals who have conducted local data quality control was 3.36 times more likely as compared to their counterparts, given the other conditions constant [AOR=3.36, 95% CI: (1.918, 5.889), p-value< 0.001]. From the total health facilities included in the study around two-third were conducted Lot Quality Assurance Sampling (LQAS). This finding was supported with a study conducted in Tigray, where health facilities conducted LQAS was 61.1% (27). This might be due to the similarity of consideration given by the government to improve data quality through supportive supervision and regular feedback.

Out of the variables which showed significant association with routine health information utilization, the expected odds of routine health information utilization among health professionals who have displayed a performance monitoring chart in their unit/department was 5.19 times more likely as compared to those who have not displayed a monitoring chart, given the other conditions constant [AOR=5.19, 95% CI: (3.177, 8.467), p-value=0.002]. This finding was supported by the study conducted in East Wollega zone (24). It is known that the presence of a performance monitoring chart in their unit/department can help them to easily capture information on the status of the unit/department and this in turn lead them to take appropriate action accordingly.

Moreover, this study exposed that the expected odds of routine health information utilization was 3.11 times higher among health professionals who had received feedback as compared to their counterparts, given the other conditions constant [AOR=3.11, 95% CI: (1.573, 6.140), p-value=0.001]. This finding is in agreement with the findings from studies done in East Gojjam zone (6), East Wollega (24) and Western Amhara. Health professionals that get feedback after supportive supervision might obtain constructive and pertinent guidance to utilize their information for improving their service delivery.

In conclusion, this study found that nearly two third of the health professionals working in the public health facilities of the south region had utilized routine health information. Place of residence, presence of HMIS personnel, assign HMIS code, overwhelming data source, use population based data, conduct regular local data quality control, provide feedback, posted performance monitoring charts and have data transfer policy had significant associations with routine health information utilization. Therefore, concerned health authorities need to work on these factors to improve the utilization.

## Limitations of the study

Our study has some limitations. Showing temporal relationship between routine health information utilization and its predictors was impossible due to the type of the study design, cross-sectional, used for the survey. The study also failed to assess routine health information utilization with other relevant variables such as health professionals' data analysis skill, attitude, governance, and availability of standard indicators in their offices. In addition, we are unable to supplement our findings with qualitative data. Moreover, the study did not include health professionals working in private health facilities due to scarcity of the resource. Conversely, the investigators have trust that the stated limitations cannot impose a significant impact on the validity of study findings.
